# The Knowns and Unknowns of Membrane Features and Changes During Autophagosome–Lysosome/Vacuole Fusion

**DOI:** 10.3390/ijms252011160

**Published:** 2024-10-17

**Authors:** Jinmeng Liu, Hanyu Ma, Zulin Wu, Yanling Ji, Yongheng Liang

**Affiliations:** Key Laboratory of Agricultural Environmental Microbiology of Ministry of Agriculture and Rural Affairs, College of Life Sciences, Nanjing Agricultural University, Nanjing 210095, China; 9221010406@stu.njau.edu.cn (J.L.); 10318103@njau.edu.cn (H.M.); 18779275370@163.com (Z.W.); yanlingji@njau.edu.cn (Y.J.)

**Keywords:** autophagic body, autophagosome, autophagosome–lysosome/vacuole fusion, double membrane, electron microscopy, lysosome, single membrane, 3D models, 2D models, vacuole

## Abstract

Autophagosome (AP)–lysosome/vacuole fusion is one of the hallmarks of macroautophagy. Membrane features and changes during the fusion process have mostly been described using two-dimensional (2D) models with one AP and one lysosome/vacuole. The outer membrane (OM) of a closed mature AP has been suggested to fuse with the lysosomal/vacuolar membrane. However, the descriptions in some studies for fusion-related issues are questionable or incomplete. The correct membrane features of APs and lysosomes/vacuoles are the prerequisite for describing the fusion process. We searched the literature for representative membrane features of AP-related structures based on electron microscopy (EM) graphs of both animal and yeast cells and re-evaluated the findings. We also summarized the main 2D models describing the membrane changes during AP–lysosome/vacuole fusion in the literature. We used three-dimensional (3D) models to characterize the known and unknown membrane changes during and after fusion of the most plausible 2D models. The actual situation is more complex, since multiple lysosomes may fuse with the same AP in mammalian cells, multiple APs may fuse with the same vacuole in yeast cells, and in some mutant cells, phagophores (unclosed APs) fuse with lysosomes/vacuoles. This review discusses the membrane features and highly dynamic changes during AP (phagophore)–lysosome/vacuole fusion. The resulting information will improve the understanding of AP–lysosome/vacuole fusion and direct the future research on AP–lysosome/vacuole fusion and regeneration.

## 1. Introduction

One of the hallmarks of macroautophagy (hereafter, autophagy) is the fusion of autophagosomes (APs) with lysosomes (vacuoles in yeast and plant cells) [1,2,3,4,5]. The defect in AP–lysosome fusion is correlated to some diseases such as human brain diseases [3,6,7,8,9]. APs are de novo generated from different membrane resources to form cup-shaped double-membrane structures [10,11,12,13,14,15,16], which then close to form immature APs. The compositions of proteins and phospholipids in APs are highly diverse, and their changes are highly dynamic [17]. Atg2 and Vps13 are critical players in phospholipid supply during AP biogenesis ([18,19,20,21] or see the review in [22]). When APs mature, they fuse with single-membrane lysosomes or vacuoles to form hybrid-membrane structures. In mammalian cells, the AP–lysosome hybrid-membrane structures are called autolysosomes (ALs) [16,23,24]. In mammalian and plant cells, APs additionally fuse with endosomes to form structures called amphisomes before fusing with lysosomes and vacuoles [25,26,27]. However, previous studies have not clarified whether amphisomes are also present in yeasts during autophagy. For simplicity and brevity, this review will omit the formation of amphisomes and the related processes, only discuss the membrane features and dynamic changes during and after AP–lysosome/vacuole fusion, and avoid focusing on the known molecules and molecular mechanisms of AP–lysosome/vacuole fusion.

The two-dimensional (2D) AP–lysosome/vacuole fusion models, especially the descriptions of membrane features at the fusion site, vary widely in the literature, with some models including incorrect single-membrane APs or double-membrane lysosomes. Therefore, identification of AP-related membranes needs to be reviewed to correctly understand them. In some models, even if the membrane feature is correctly described, the fusion process is incorrect. Furthermore, the fusion and post-fusion issues are at least three-dimensional (3D); therefore, many details are not able to be demonstrated with 2D models. We introduced 3D models to better understand the knowns and unknowns of membrane changes and used a mathematical method to estimate and emphasize the rapid membrane changes and regeneration during fusion and post-fusion processes. We think the information in this review is important and will direct the future research regarding AP–lysosome/vacuole fusion in some degree in the autophagy field.

## 2. Different Electron Microscopy Methods Observed Double-Membrane APs and Single-Membrane Lysosomes/Vacuoles

**Figure 1:**
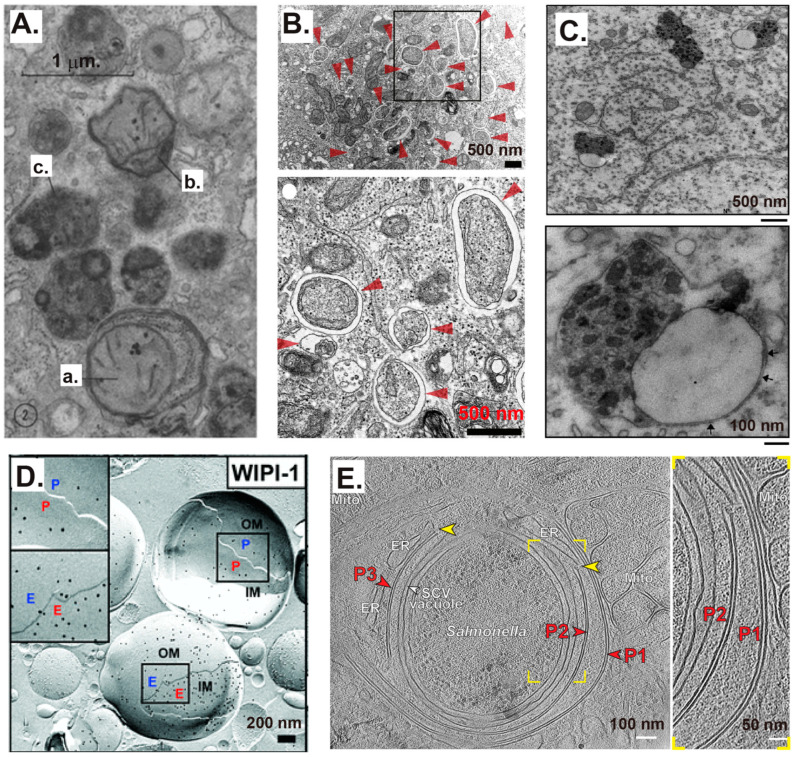
Representative autophagosome (AP)-related ultrastructures in mammalian cells observed by electron microscopy (EM) in the literature. (**A**) The first study to show AP-related structures using conventional transmission electron microscopy (TEM) of perfused rat liver treated with glucagon. This panel is modified from Figure 2 published in a previously published study [29], and has been represented with permission from the Rockefeller University Press. Body a was interpreted as a rather early form of a lysosome that included mitochondria and rough endoplasmic reticulum (ER) within its boundaries, although it is more like an AP with double membranes. Bodies b and c were more likely autolysosomes (ALs). (**B**) Conventional TEM showing APs with obvious double membranes from siStx17-treated HeLa cells cultured in starvation medium. APs are indicated by red arrowheads. This panel is from Figure 4D(c,d) in a previously published study [31], and has been represented with permission from Elsevier. (**C**) Representative electron micrographs of incompletely fused APs and lysosomes in a pyramidal neuron from a S367A knock-in mouse. The bottom picture shows a higher magnification of an AL in the process of fusion. Arrows indicate the double membrane of the unfused AP. This panel is from Figure 1A,B of a previously published study [32], and has been represented with permission from the National Academy of Sciences. (**D**) Freeze-fracture immuno-EM images showing the double membrane of APs induced by nutrient starvation in unfixed stable GFP-WIPI-1 U2OS cells. WIPI-1 was identified with anti-GFP on both sides of the monolayers of the inner and outer AP membranes. Monolayers were termed protoplasmic (P)- and extracellular (E)-faces according to the P-face of the outer membrane (OM) facing the cytoplasm. This panel is from Figure 1C in a previously published study [33], and has been represented with permission from John Wiley and Sons. (**E**) The in situ correlative cryo-electron tomographic (cryo-ET) slice highlights a double-membrane phagophore’s expansion (P1) on top of the existing phagophore (P2). The enlarged view highlights the distance between P1 and P2. Yellow arrowheads indicate contact sites between the phagophore rim and the ER. The mCherry-GAL8-expressing HeLa cells were infected with GFP-expressing Salmonellae and analyzed at 1.5 h post-infection. This panel is from Figure 2D in a previously published study [35], and has been represented with permission under CreativeCommons Attribution-NonCommercial-NoDerivativesLicense 4.0 (CC BY).

Conventional transmission electron microscopy (TEM) is the gold standard and the most sensitive method to “see” AP-related membranes. APs are defined as double-membrane-bound compartments containing cytoplasmic materials and/or organelles in TEM images. However, correct interpretation of electron microscopy (EM) images requires clear pictures and special expertise; otherwise, other compartments may be incorrectly identified as AP compartments [28]. The first description of AP-related structures was reported in 1962 by Ashford and Porter ([29] and Figure 1A). Body a was interpreted as a lysosome enclosing the mitochondria, rough ER, and other organelles. Bodies b and c are more advanced structures than Body a. However, the lysosomal membrane and the supposed remnant membranes of the APs are not very clear in these bodies.

In TEM graphs in the early literature, some reported APs in mammalian cells seem to have only one electron-dense limiting membrane, whereas others appear to consist of two or several separate membranes. Occasionally, the limiting membrane may not show contrast at all. These differences may probably be attributable to differences in the cell types, culture durations, and fixation and sample-preparation methods, such as lipid extraction during sample preparation [28]. Therefore, identification of AP-related structures using TEM often requires other supporting methods, such as fluorescence observation and immuno-TEM. Correlative light and electron microscopy (CLEM) can perfectly resolve the analysis of the same specimen using a combination of light and electron microscopy tools [30]. Established and novel techniques have gradually promoted the observation of AP-related structures in mammalian cells. For example, APs with clear double membranes were observed by conventional TEM analysis of HeLa cells treated with siStx17 and cultured in starvation medium for 1 h ([31] and Figure 1B). The typical features of AP–lysosome fusion at the intermediate stage, with the non-fused single membrane of lysosomes on one side, the non-fused double-membrane of autophagosomes on the other side, and the remnant AP inner membrane (IM) and the lysosome–AP outer membrane (OM) fusion membrane in the middle, were clearly observed in the brains of PS1-S367A mice ([32] and Figure 1C).

The combination of freeze-fracture and immuno-EM can be used for three-dimensional (3D) analysis of APs and their features. The OM and IM of APs with WD-repeat protein interacting with phosphoinositides 1 (WIPI-1) on both the protoplasmic (P)- and extracellular (E)-faces of monolayers were observed in unfixed stable green fluorescence protein (GFP)-WIPI-1 U2OS cells induced by nutrient starvation using Earle’s balanced salt solution (EBSS) for 6 h ([33] and Figure 1D). The emerging cryo-focused ion beam (FIB) and cryo-electron tomography (ET) techniques can help efficiently visualize AP-related membrane structures, providing new insights to clarify autophagy processes [34]. The cryo-FIB and cryo-ET techniques have revealed clear double-membrane phagophores (also called isolation membranes or unclosed APs) that could progress to form new APs. Furthermore, multiple phagophores with clear double membranes have been observed, with a double-membrane phagophore expansion (P1) on top of an existing phagophore (P2) ([35] and Figure 1E). Thus, the double-membrane characteristics of phagophores or APs in mammalian cells have been verified using multiple EM methods, although the existence of multiple layers of phagophores or APs cannot be ruled out.

**Figure 2:**
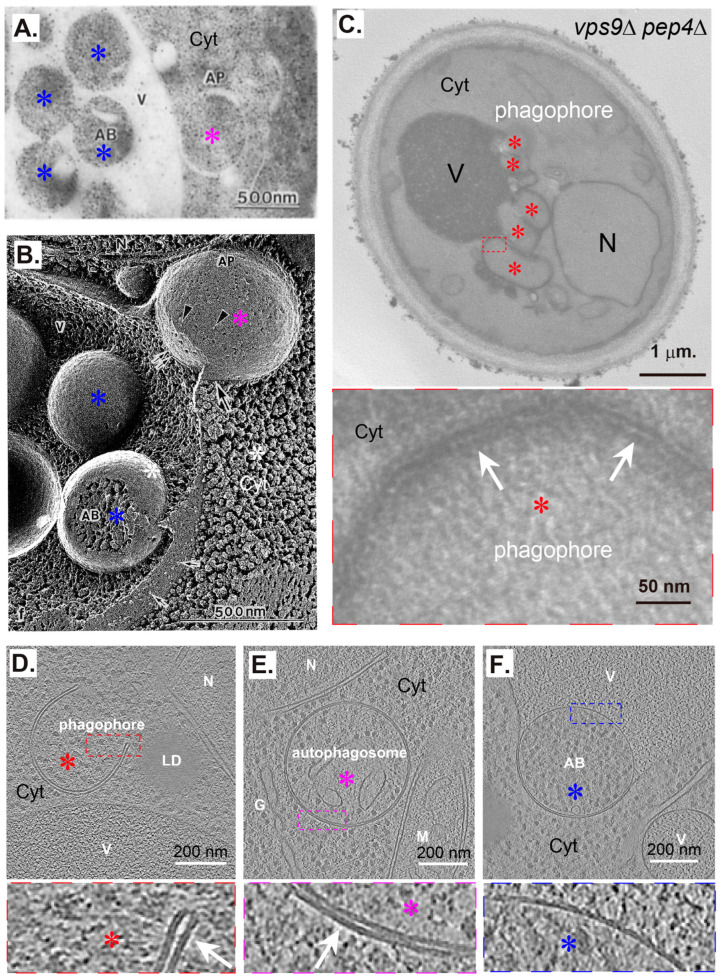
Representative AP-related ultrastructures in yeast cells observed by EM in the literature. (**A**) Immunoelectron microscopy (Immuno-EM) demonstrated the cytosolic enzyme alcohol dehydrogenase inside the double-membrane AP structure in the cytosol and inside the single-membrane autophagic bodies (ABs) in the vacuoles of yeast cells incubated in synthetic medium with 2% glycerol (SG) as the carbon source. This panel is modified from Figure 3a in a previously published study [37], and has been represented with permission from the Rockefeller University Press. (**B**) Freeze-etching method showed the fusion between the AP and vacuole in unfixed yeast cells incubated in synthetic defined medium that lacks nitrogen and amino acid (SD-N) medium. The large arrow indicates the OM of the AP. The double arrow indicates the inner membrane (IM) of the AP. Arrowheads indicate intramembrane particles on the autophagosomal membrane. Small arrows indicate intramembrane particles on the vacuolar membrane. This panel is modified from Figure 4f in a previously published study [38], and has been represented with permission from the Japanese Society of Cell Biology. (**C**) Conventional TEM shows a cluster of phagophores with double membranes in the cytosol in *vps9Δpep4Δ* cells starved in SD-N medium. This panel is modified from the right column in Figure 4C from a previously published study [39], and has been represented with permission under an Attribution–Noncommercial–Share Alike 3.0 Unported Creative Commons License (http://creativecommons.org/licenses/by-nc-sa/3.0, accessed on 3 October 2024). (**D**–**F**). Exemplary tomogram slices and segmentations of key autophagy steps captured with correlative cryo-ET from yeast cells starved in SD-N. (**D**) A cup-shaped phagophore with double membrane in the cytosol. (**E**) A double-membrane AP in the cytosol. (**F**) A single-membrane autophagic body (AB) in the vacuole just after fusion. The membrane features in the frames in (**D**–**F**) were amplified to show details. These three panels are from Figure 1F–H in a previously published study [40], and have been represented with permission under CreativeCommons Attribution-NonCommercial-NoDerivativesLicense 4.0 (CC BY). The AP-related structures in this figure were labeled with red ∗ for phagophores, purple ∗ for APs, and blue ∗ for ABs.

The findings for the membrane features of AP-related structures in yeast cells were similar, although the first report of yeast autophagic bodies (ABs) appears almost 30 years after that of mammalian cells. The first description of the morphology of ABs in vacuoles was demonstrated in a proteinase-deficient yeast mutant using conventional TEM [36]. Double-membrane APs in the cytosol and single-membrane ABs in vacuoles were further detected by conventional TEM ([37], and Figure 2A), although the membrane features of double and single membranes are difficult to distinguish by non-experts. The freeze-etching method revealed the fusion between the AP OM and vacuole membrane in yeast cells incubated in synthetic defined medium that lacks nitrogen and amino acid (SD-N) medium for 2.5–3.5 h ([38], and Figure 2B). When we studied the phagophores in Vps21 module mutants, we observed double-membrane characteristics of yeast phagophores in *vps9Δpep4Δ* cells ([39], and Figure 2C). The double-membrane features of yeast phagophores and APs in the cytosol and single-membrane ABs inside the vacuoles were clearly demonstrated using the cryo-FIB and cryo-ET methods ([40,41], and Figure 2D–F).

While APs and phagophores may vary in layers in mammalian cells, they consistently show two layers in yeast cells. For the earlier TEM graphs in which the membrane features could not be distinguished clearly, the membranes may need to be revisited with advanced and reliable new EM methods to confirm the early findings. In general, these methods indicate that APs and phagophores are double-membraned, whereas lysosomes/vacuoles are single-membraned, findings that most researchers in the field of autophagy agree upon.

## 3. Examples of Incorrect or Problematic AP-Lysosome/Vacuole Fusion 2D Models in the Literature

**Figure 3:**
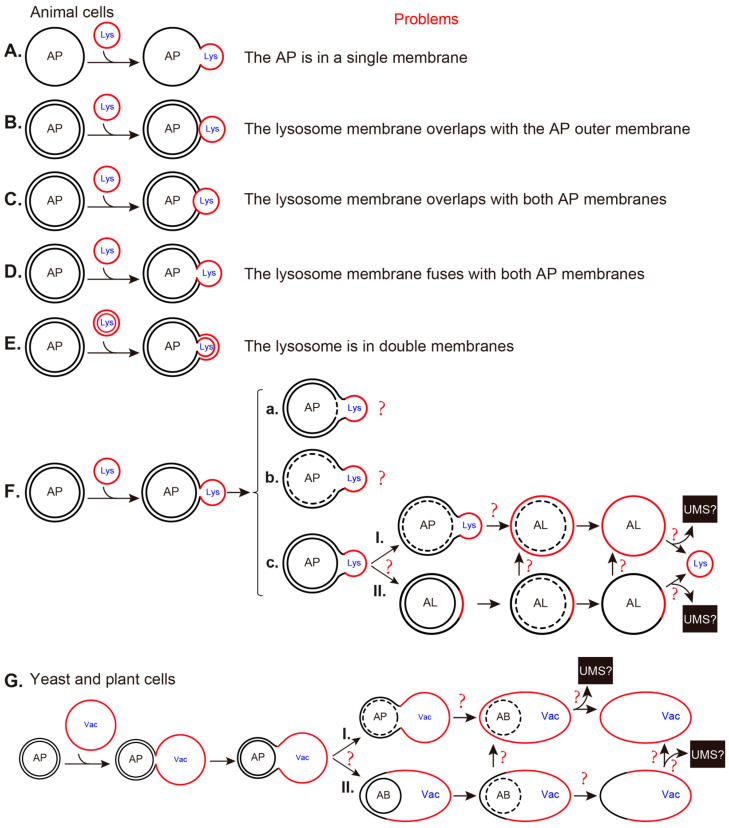
Examples of incorrect or problematic AP–lysosome/vacuole fusion models excluding amphisomes in two dimensions (2D) in the literature. For simplicity, only membranes of one AP and one lysosome/vacuole were drawn with circular lines. Red circular lines are for lysosomal/vacuolar membranes and black circular lines are for AP membranes. Dashed lines are for disrupted membranes. AP: autophagosome; Lys: lysosome; Vac: vacuole; AB: autophagic body; UMS: unknown membrane structure. (**A**–**E**) Incorrect AP–lysosome/vacuole fusion models in animal cells. The main problems are indicated at the right column. (**F**,**G**). Problematic AP–lysosome/vacuole fusion models in animal cells (**F**) and in yeast and plant cells (**G**). The derivative possible models are further categorized as a–c, then c further as I–II (**F**) or directly as I–II (**G**). The unclear or uncertain processes are highlighted with “?” marks. The figure contents were further discussed in detail in the main text and partially in a recent paper [42].

Many models have been used to illustrate AP–lysosome/vacuole fusion and post-fusion issues in the literature. We summarized and categorized these models in a simple manner, drawing the fusion of one AP to one lysosome/vacuole in two dimensions (Figure 3). In models A–E, different errors occur, including drawing single-membrane AP (A) and double-membrane lysosomes (E), or no membrane fusion (B–C) or wrong membrane fusion (D). We provided detailed explanations for these wrong models in our recent Letter to the Editor of Autophagy [42].

In model F, the single membrane of a lysosome fuses with the OM of an AP to open a channel. When the channel expands, the lysosomal contents move forward towards the AP side. These partially or fully fused compartments are known as autolysosomes (ALs) [23,24]. Different assumptions have been made regarding the subsequent changes in the AP IM: a. the AP IM near the channel is broken to allow the lysosomal contents to enter the AP, or b. the AP IM near the channel disappears, and the remaining AP IM is gradually digested. Figure 1C indicates that situations in a and b would not occur because the AP IM next to the lysosomal contents seems intact. The situation in c most likely fits the results shown in Figure 1C. After the lysosomal membrane is fused with the AP OM, two main routes can often describe changes in the membranes. In route I, during the fusion, the digestion of the AP IM with contents is initiated, and the hybrid membrane of the AP OM and the lysosomal membrane are mixed and homogenized to form a unique membrane with similar composition to a lysosomal membrane. When the AP IM with its contents is completely digested, the final AL is reformed into lysosomes and possible unknown membrane structures (UMSs). In route II, the lysosomal membrane fuses with the AP OM at the contact sites and is flattened to achieve the same curvature as that of the neighboring AP OM. Thus, the fused lysosomal membrane consists only of a portion of the entire AL membrane and is located around the fusion site. Then, the lysosomal contents from the fused lysosomes enter the AP intermembrane space (the space between the outer and inner membranes of the AP) until the lysosomal contents wrap the entire AP IM. This pattern is supported by the observation that ring-shaped LysoTracker Red structures appeared on the STX17-positive AP structures at the early stage and gradually appeared inside the AP lumen, indicating that immediately after fusion, lysosomal contents existed only in the space between the outer and inner membranes of APs but not inside APs [43,44]. The AP IM is expected to break in the later stages. When the digestion of the AP IM with its contents is initiated until complete digestion is achieved, the final AL is reformed into lysosomes and possible UMSs. Meanwhile, the hybrid membrane may be converted to lysosomal membrane-like membranes during or after digestion of the AP IM with its contents. Through routes I or II, the surface area of the autolysosomal membrane should be larger than that of the original AP OM because of the received lysosomal membrane. The possibilities and potential problems in these routes will be discussed further using 3D models in the next figure.

The size of APs relative to vacuoles in yeast and plant cells is opposite to that of APs relative to lysosomes in mammalian cells [45]. AP–vacuole fusion is supposed to be similar to AP–lysosome fusion, but with variations. In model G, the single membrane of the vacuole fuses with the OM of the AP to open a channel so that the vacuolar contents can enter the AP intermembrane space until finally wrapping the ABs, as shown in Figure 2F. After the fusion, two similar routes can describe the changes in the membranes as lysosome–AP fusion, except that the final vacuole directly originates from the hybrid structure after AB is completely digested or it needs an additional membrane mixture step. In route II, the hybrid AP OM–vacuole membrane contained a small area of AP OM and a large area of the vacuolar membrane. Whether and how these different membranes would mix and homogenize to form vacuolar membranes before regeneration is unknown. When multiple APs are fused with the same vacuole, the selection of a subsequent fusion site and membrane transformation, including regeneration, is more complex and has not been discussed in the literature. These issues are further discussed in the following 3D models in the next figure.

## 4. 3D Models Help Us Understand AP–Lysosome/Vacuole Fusion and Post-Fusion Issues

**Figure 4:**
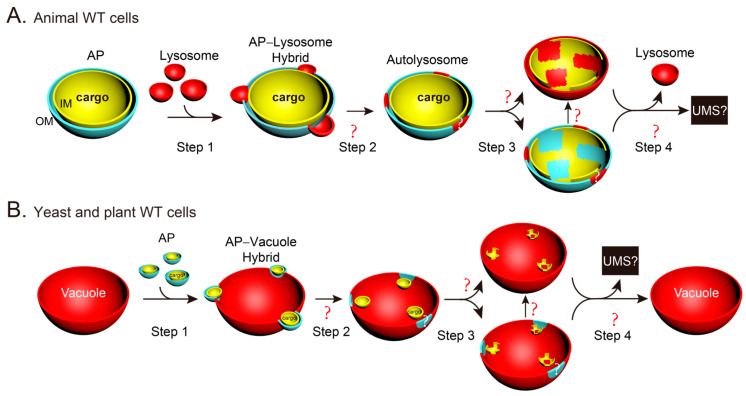
Three-dimensional (3D) illustrations showing the possible spatiotemporal changes in the membranes during and after the fusion of an AP with a lysosome/vacuole. The drawings are based on the promising key steps outlined in Figure 3F,G and divided into four key steps. (**A**) In mammalian WT cells, the steps can be summarized as follows: Step 1, the membranes of the single-membrane lysosomes fuse with the OM of the double-membrane AP to form an AP–lysosome hybrid structure. Step 2, the lysosomal contents invade the AP intermembrane space, and the fused lysosomal membrane may flatten to achieve the same curvature as the remaining neighboring AP OM or are already mixed and homogenized with the remaining neighboring AP OM, and the lysosomal hydrolases may start to destroy the AP IM and its cytosolic contents at an uncertain time. Step 3, the AP IM structures and the cargoes inside the autolysosomal membrane are destroyed by lysosomal hydrolases while the lysosomal membrane is already mixed well with the AP OM (upper row) or are still located at a certain narrow area (lower row). Step 4, unclear membrane dynamic changes and site selections occur to generate new lysosomes and possible unknown membrane structures (UMSs) from the AL. (**B**) In yeast and plant WT cells, the steps can be summarized as follows: Step 1, the membrane of the big single-membrane vacuole fuses with the OMs of the small double-membrane APs to form a hybrid structure. Step 2, the ABs enter the vacuolar lumen and are located near their AP OMs, while the remaining AP OMs are still located in a certain narrow area or already mixed well with the vacuolar membrane, and the vacuolar hydrolases may start to destroy the ABs at an uncertain time. Step 3, the ABs are destroyed by the vacuolar hydrolases and disappear, while the hybrid membrane is as uniform as the vacuolar membrane (upper row) or non-uniform with some AP OMs (lower row). Step 4, unclear membrane dynamic changes and site selections occur to generate a new vacuole with a homogeneous membrane and possible UMSs. The unclear or uncertain processes are indicated with “?” marks.

The AP–lysosome/vacuole fusion process is at least a 3D process with spatiotemporal changes. The 2D models are not able to reflect spatial changes even if the membrane features and fusion process are correct. When the AP–lysosome/vacuole fusion process is displayed in 3D models, the changes in the membrane surface area (perimeter length in 2D models) and fusion region (contact sites in 2D models) can be more easily understood. In mammalian cells, multiple lysosomes fuse with the same AP ([46], Movie 1). These lysosomes are most likely to sequentially fuse with the AP, although the possibility that some lysosomes simultaneously fuse with the AP cannot be ruled out. Regardless of the pattern, the cells need to select the fusion sites, and previous studies have not clarified whether the same or different regions or sites are selected for fusion. We assumed that the cells would choose different sites to fuse with different lysosomes and analyzed the possible dynamic changes in the membranes in a 3D model (Figure 4A). After the single membrane of a lysosome fuses with the OM of an AP to form an AP–lysosome hybrid structure (also called an AL), a channel or a hole allows the lysosomal contents to access the intermembrane space between the outer and inner membranes of the AP. The fused lysosomal membrane is supposed to bend to reach the same curvature as its neighboring AP OM, although evidence for this is lacking. Different lysosome–AP fusions can undergo similar steps. Either before or after the lysosomal contents cover the whole surface of the AP IM, the lysosomal hydrolases may degrade the AP IM with its cytosolic contents at unidentified times and through unidentified mechanisms. The hybrid membrane may be converted to a lysosome-like membrane during or after digestion of the IM with cargoes. When autophagy is terminated, new lysosomes are reformed from ALs through the reactivation of mammalian target of rapamycin [46]. The autolysosomal component recycling (ACR) complex is involved in autophagic lysosome reformation (ALR) [47,48]. However, the surface location of the AL that is selected to reform lysosomes remains unclear, especially when ALs with uniform or non-uniform membranes are used to generate lysosomes. In addition, the existing literature does not clarify whether proteins and lipids are recycled together to form membrane patches or separately as components. If the phospholipids in ALs are recycled separately, what are their destinations? Furthermore, the existing literature does not clarify whether the entire AL is converted to new lysosomes or some parts are converted to other UMSs.

In yeast and plant cells, vacuoles are usually larger than APs and multiple APs often fuse with the same vacuole [45]. Therefore, the double-membrane APs could enter the vacuolar lumen to become single-membrane ABs through the fusion of the AP OMs with the vacuolar membrane. The dynamic changes in the membranes during AP–vacuole fusion are illustrated in 3D (Figure 4B). This fusion process requires the selection of fusion sites on the vacuolar membrane and the bending of the AP OM to achieve the same curvature as its neighboring vacuolar membranes. The ABs wrapped by vacuolar liquid are degraded by vacuolar hydrolases and recycled to the cytoplasm for reuse. The dynamic changes in the hybrid membrane, especially after multiple APs are fused to the same vacuole, have not been studied in detail. If fusions preferentially occur on the same site (area), the next fusion may occur between the OM of a new AP and the remaining OM of an old AP connected to the vacuolar membranes from the previous round of fusion. This process may occur repeatedly. Considering the fluidity of the membranes, the old AP membrane that had fused previously is not likely to have remained in the same location on the vacuolar membrane. However, how quickly or distantly membrane fluidity occurs under such conditions remains unknown. Therefore, the hybrid membrane may be uniform or non-uniform during AB digestion. At the end of these processes, how do the OMs of APs connected to vacuolar membranes convert to vacuole membranes or UMSs? If each fusion occurs at a new spot/location in the vacuolar membrane, how is the previous AP–vacuole hybrid membrane spatiotemporally regulated for reconstruction after fusion? How are the second and third fusions, until multiple rounds of fusion, spatiotemporally regulated?

In yeast cells with defective vacuolar proteases, ABs would gradually accumulate in vacuoles along with autophagy induction and finally fill the whole vacuolar lumen [36]. Since the AP OMs fuse to the vacuolar membrane, these fusion processes cause the vacuole surface area to gradually increase unless the vacuolar membranes are rapidly regenerated. The expansion of vacuoles could be directly observed with TEM and the increase in vacuolar volumes and surface areas could be calculated (estimated) with TEM graphs. We used an example of published AB accumulation in *pep4Δ* cells during autophagy to discuss the potential membrane changes during AP–vacuole fusion as described in detail below.

## 5. The Fusion of AP Outer Membrane to Vacuole Significantly Increases Vacuole Volumes and Surface Areas

**Figure 5:**
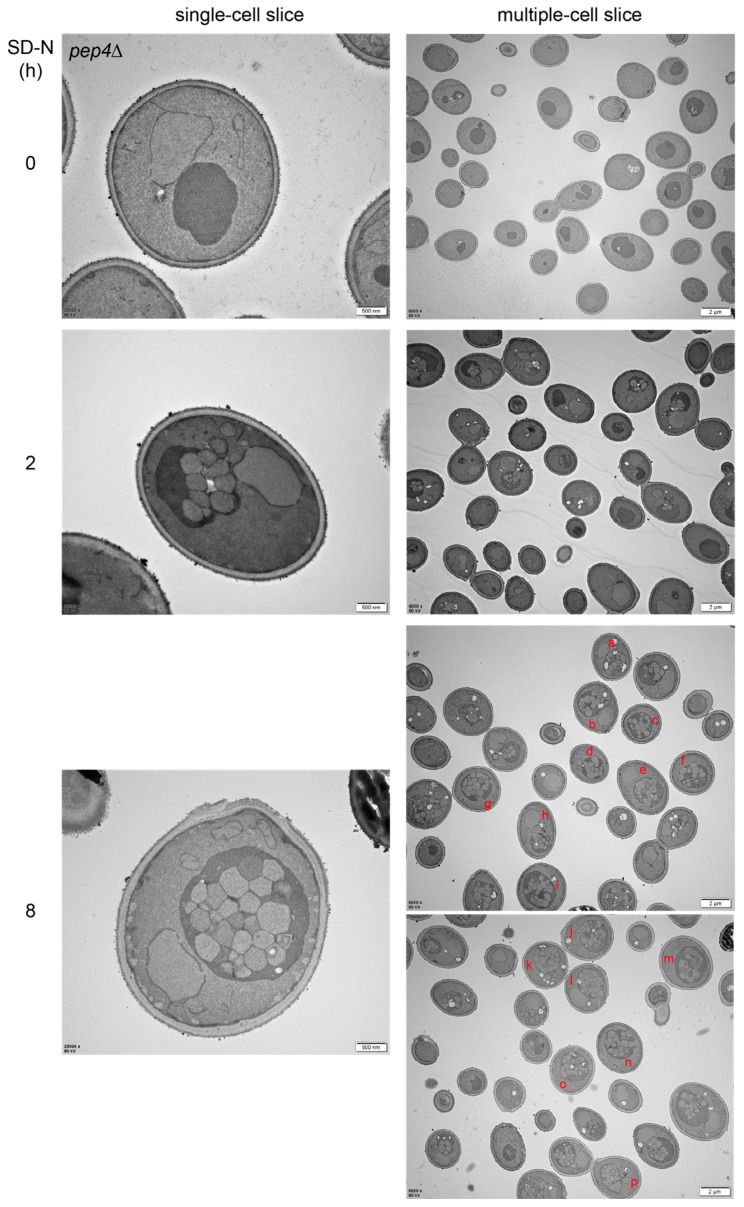
Exemplary images of the accumulation process of ABs in the vacuoles of *pep4Δ* cells during nitrogen starvation. These images are the raw data in the bottom row of Figure 3A in a previous study [41] and similar data have been published in Figure 1 in another study [36]. TEM graphs for the ultrastructure of *pep4Δ* cells starved in SD-N medium at the indicated durations. Representative graphs for single-cell and multiple-cell slices are shown. The planes marked by red letters (a–p) were subjected to the measurement of the dimeters of ABs and vacuoles for quantifying AB numbers and contributed surface area to vacuolar membranes through the Kepler conjecture as described in the main text.

Autophagy during nitrogen starvation in yeast cells before termination is a continuous process, during which APs continue to fuse with the same vacuole, as shown by a time-lapse video in wild-type cells [39]. The ultrastructural data of *pep4Δ* cells starved in SD-N for different durations (Figure 5) could be analyzed to obtain more information on the changes in vacuole volume and surface area. When the *pep4Δ* cells were starved in SD-N, besides the gradual accumulation of ABs in the vacuoles, the vacuole diameter also gradually increased from approximately 1 µm at 0 h to 1.7 µm at 8 h in averaged measurements obtained using approximately 30 cell slices for each time duration. On the basis of the surface area and volume calculation equations for a ball (see below), the vacuole surface area increased approximately (1.7/1)^2^ = 2.89-fold, and the vacuole volume increased about (1.7/1)^3^ = 4.913-fold. According to the Kepler conjecture, the maximum space of equally sized small spheres filling and packing a three-dimensional big spherical space was approximately 74.05%, which was approved by Thomas Hans [49]. Therefore, if the diameter of a large sphere space is *D* = 2*R*, the diameter of a small sphere is *d* = 2*r*, and the number of small spheres filling the large sphere is *n*, then

$$\frac{4}{3}\pi r^{3}\times n=74.05\%\times \frac{4}{3}\pi R^{3}$$

If the AB accumulation in vacuoles did not reach the maximum of 74.05%, but was approximately 50%, then

$$\frac{4}{3}\pi r^{3}\times n=50\%\times \frac{4}{3}\pi R^{3}$$

$$r^{3}\times n=50\%\times R^{3}$$

$$n=50\%\times R^{3}/r^{3}$$

$$n=50\%\times {\left(\frac{D}{2}\right)}^{3}/{\left(\frac{d}{2}\right)}^{3}$$

$$n=50\%\times \frac{D^{3}}{d^{3}}$$

Through measuring the diameters of 148 ABs in the vacuoles of 17 cell slices of *pep4Δ* cells starved in SD-N for 8 h, the average diameter of the small spheres (ABs) (*d*) was approximately 0.347 ± 0.04 µm. With the measured diameter of the large sphere (each vacuole) (*D*) and the calculated average diameter of ABs in each slice (*d_ave_*), the number (*n*) of ABs filling each vacuole could be calculated (estimated) as follows:

$$n=50\%\times \frac{D^{3}}{{\left(d_{ave}\right)}^{3}}$$

With the estimated number (*n*) of ABs in each vacuole, the average AB diameter of 0.347 µm, and the measured diameter of each vacuole (*D*), the total volume and surface area of ABs in each vacuole and the volume and surface area of the vacuole could be easily calculated as follows:

$$V_{vac}=\frac{4}{3}\pi R^{3}=\frac{4}{3}\pi {\left(\frac{D}{2}\right)}^{3}\mathrm{or}\;V_{ABs}=n\times \frac{4}{3}\pi r^{3}=n\times \frac{4}{3}\pi \times {\left(\frac{d_{ave}}{2}\right)}^{3}$$

$$S_{vac}=4\pi R^{2}=4\pi {\left(\frac{D}{2}\right)}^{2}\mathrm{or}\;S_{ABs}=n\times 4\pi r^{2}=n\times 4\pi \times {\left(\frac{d_{ave}}{2}\right)}^{2}$$

It was found that the calculated total volume occupied by ABs in each vacuole ($V_{ABs}$) was approximately 59.4 ± 2.9% of the measured volume of that vacuole ($V_{vac}$), which was very close to the setting of approximately 50% of the vacuole volume filled by ABs. In this situation, the total surface area of the filled ABs in each vacuole ($S_{ABs}$) was 3.04 ± 0.42 folds to the measured surface area of the vacuole ($S_{vac}$). While some of the contributing membranes from the fused AP OMs are used to expand the vacuole surface area to increase to the final 2.89 folds, most of the other contributing membranes from the fused AP OMs are removed from the current vacuolar surface; otherwise, the supposed vacuolar surface area would be approximately 3-fold greater than the current vacuolar surface area at 8 h with SD-N treatment.

Vacuoles change their morphology through various processes, including vacuolar fusion, fission, and invagination, under normal growth conditions or in response to stress. However, studies on how cells control the vacuolar size during autophagy are limited. Vacuolar invaginations that form autophagic tubes through microautophagy have been proposed to play a role in the homeostasis of the vacuolar membrane size during macroautophagy [50]. In the following section, we discuss the possible contributions of vacuolar invaginations and other approaches to control vacuolar size and volume during membrane reconstruction or regeneration after AP–lysosome/vacuole fusion.

## 6. Other Unanswered Questions Behind Normal AP–Lysosome/Vacuole Fusion

Figure 4 illustrates the major events during AP–lysosome/vacuole fusion in three dimensions and Figure 5 in combination with the Kepler conjecture calculation indicates that the fusion theoretically contributes to vacuolar surface area and volume significantly but the actual retention of the membrane is much lower. Therefore, the contribution of the AP OM to the hybrid membrane and the regeneration of membranes from the hybrid structure during fusion and post-fusion should be highly dynamic. In addition to the questions asked during the interpretation of these models in Figure 4, there are many other specific questions that need to be addressed below.

(1) What is the signal to trigger fusion when APs become mature? In budding yeast, after the phagophores are closed to become immature APs, most Atg proteins and phosphoinositol 3-phosphate (PI3P) on the OMs of immature APs are cleared, which is followed by maturation of APs [51,52,53]. Subsequently, the OM of a mature AP is believed to fuse with the vacuolar membrane. The IM and its contents (also called an AB) are delivered to the vacuolar lumen for degradation and recycling. However, the signal that triggers fusion when APs mature remains unclear. Recruitment of the autophagosomal SNARE protein STX17 has been suggested to trigger fusion in mammalian cells [31]. Ykt6, a SNARE protein, is involved in autophagosome–vacuole/lysosome fusion [54,55,56]. Recently, STX17 was found to be recruited to mature APs by electrostatic interactions between the positively charged C-terminal region of STX17 and the negatively charged autophagosomal membrane owing to the accumulation of phosphatidylinositol-4-phosphate (PtdIns4P) on the surface of mature APs [57,58]. However, the signal that directs the localization of Ykt6 to the OMs of completed APs, but not to phagophores, remains unknown. The other factors that trigger fusion and the upstream signal that regulates PtdIns4P accumulation are also not known. In addition, what is the signal that triggers the fusion between lysosomes/vacuoles and phagophores mentioned at the end of this section?

(2) What are the contents between the OMs and IMs of APs? The space between the two AP membranes appears to be “empty-lucent” during standard EM procedures. Different sources of autophagosomal membranes have been reported, including the mitochondria, ER–mitochondria contact sites, Golgi, plasma membrane, endosomes, ER–Golgi intermediate compartment, and COPII vesicles [10,11,12,13,14,15,16]. Immuno-EM showed that the COPII protein Axl2 was distributed not only on autophagosomal membranes but also in the space between the outer and inner membranes of APs, indicating that COPII vesicle components are at least possible components of the contents between the OMs and IMs of APs, although the details of conversion/merging of COPII vesicle membranes to the pre-existing autophagosomal membranes are lacking [16]. The potential presence of additional components between the OMs and IMs of the APs is also unknown.

(3) Are the sites for fusion selective for both lysosomes/vacuoles and APs? During autophagy, “fixed” phagophore assembly sites (PASs) are present next to the vacuoles in yeast cells [59,60]. During maturation, APs are thought to lose contact with vacuoles [61]. Whether selective and fixed sites for fusion exist on lysosomes/vacuoles and APs remains unknown. The fusion sites/areas on vacuoles appear to be restricted to a specific area, since the phagophores (unclosed APs) are clustered outside the vacuoles at the nucleus–vacuole junction site instead of being randomly distributed on vacuolar membranes in Vps21 or endosomal sorting complex required for transport (ESCRT) mutant cells [39,52,53]. However, whether yeast cells use PASs for AP fusion is unknown, and the presence of specific sites on mammalian APs and lysosomes that are used for fusion also remains unknown.

(4) What are the spatiotemporal changes of the AP–lysosome/vacuole hybrid membrane during fusion? When the AP OM and lysosomal/vacuolar membrane fuse, the fusion at the contact site supposedly forms a hole or channel. During vacuole–vacuole fusion, SNARE-mediated membrane fusion is arrested at pore expansion to regulate the volume of an organelle and osmotic pressure gradients, providing a simple mechanism to rapidly adapt the organelle volume to increase its content. Membrane continuity of lipids and proteins is thought to be rapidly re-established after membrane fission during vacuole–vacuole fusion [62]. The vacuole/lysosome may adapt to the increasing autophagosomal input during AP–lysosome/vacuole fusion because the volume also increases with osmotic pressure gradients.

As shown in Figure 1C and Figure 2F, the fusion hole has already expanded wide enough to allow the AP IM with cytosolic contents (called AB in yeast and plant cells) to enter the lysosomal/vacuolar lumen. However, in these intermediate hybrid states, the compositions of the non-fused AP OMs and non-fused lysosomal/vacuolar membranes should be quite different from those of the fused AP–lysosome/vacuole membranes. The spatiotemporal changes in the fused part and the subsequent changes in the non-fused part are unclear. At least two major possibilities have been suggested for the spatiotemporal changes in AP–lysosome/vacuole hybrid membranes (Figure 4): (a) a continuous step with simultaneous membrane fusion and mixing from the beginning to the end; and (b) discrete steps with fusion and mixing at the contact sites to form a hole or channel first, followed by some pause for fusion and mixing until the lysosomal/vacuolar contents completely surround the AP IM to initiate further fusion and mixing, although this initiation may occur at intermediate time points. To date, these questions have remained unanswered. Therefore, the spatiotemporal changes in the AP–lysosome/vacuole hybrid membrane during fusion, including the changes in the size and volume of the hybrid structure, remain unclear.

(5) Are some forces needed to separate the IM from the OM of an AP during AP–lysosome/vacuole fusion? To date, no such determinations have been made. Freeze-fracture EM and other studies have shown that the two membranes of an AP contain relatively few transmembrane proteins, most of which are located almost exclusively on the OMs of the APs. This implies that these membranes are neither connected nor tethered to each other ([63,64] or see the review [65]). This also causes the characteristic appearance of APs in the EM images; the two membranes are easily separated from each other, making the space between the two membranes appear “empty-lucent” in standard EM procedures. It remains unknown whether the limited particles on the membranes are membrane proteins such as Atg9/ATG9A and SNARE proteins with transmembrane domains, as reported by other methods [31,66,67]. Most likely, the osmotic pressure gradients from the liquid lysosomal/vacuolar contents drive the separation during AP–lysosome/vacuole fusion [62].

(6) Do APs enter vacuolar lumens or do lysosomal contents enter AP intermembrane spaces? These situations have been previously addressed in detail [45], but the differences between AP–lysosome and AP–vacuole fusion require emphasis. In yeast and plant cells, APs are usually smaller than vacuoles. Therefore, multiple APs would enter the same vacuolar lumen in the AB format for degradation and recycling. However, in mammalian cells, based on both published data obtained using fluorescence and immuno-EM images, the AP size is usually larger than the lysosome size [45]. Generally, ABs do not form in higher eukaryotes other than plants, and in some rare cell types in other species [45]. In contrast, lysosomal contents are supposed to enter the space between the OMs and IMs of APs before destroying the IMs of APs to further digest the AP contents. Multiple lysosomes fuse with a single AP (Movie 1 in [46]). The number of lysosomes that need to fuse with an AP to digest it, the number of APs that can be digested by the same competent lysosome, and the presence of sequential fusions between the newly formed AL and APs are all unclear. In either situation, the lysosomal membranes and AP OMs would fuse and mix to form the AL membrane. Therefore, the lysosomal contents and the AP IMs with contents would become the AL contents, which are supposed to be digested by lysosomal enzymes [43].

(7) Why do the lysosomal/vacuolar hydrolases destroy the IM of an AP and its contents but not the hybrid AP–lysosome/vacuole membrane? The lysosomal/vacuolar membrane is not destroyed by its own hydrolases. Lysosomal membranes contain abundant heavily glycosylated proteins that are thought to protect lysosomal membranes from lysosomal hydrolases [68]. For mammalian APs that are directly fused with lysosomes, the OMs need to have features that are different from those of the IMs to protect them from digestion by lysosomal hydrolases. If the AP OM is gradually converted to a lysosomal/vacuolar membrane during fusion, the hybrid membrane may be protected from lysosomes/vacuoles. Observation of intramembrane particles in mammalian organelles using freeze-fracture EM indicates that AP OMs may mix with endosomes to form amphisome membranes, which may further mix with lysosomal membranes to form AL membranes [64,69]. Immuno-EM has also shown that endosomes or lysosomes containing lysosome-associated membrane glycoprotein 1 (LAMP1) mix with the AP membranes of both closed and unclosed APs, but differently [44], although the spatiotemporal regulation of the membrane mixture remains unknown. The aberrant fusion of a phagophore with lysosomes/late endosomes results in the distribution of glycosylated LAMP1 throughout the phagophore membrane, limiting access to lysosomal proteases and lipases, and thus impairing degradation [44]. These membrane features may explain why lysosomal contents destroy the IMs but not the fused OMs of APs. In budding yeast, freeze-fracture analysis also showed that the vacuolar membrane is transmembrane-particle-rich, and intramembrane particles are present on the autophagosomal membrane, whereas ABs in the vacuolar lumen do not contain transmembrane particles [38,50]. The phenomenon occurring in mammalian cells may occur in yeast and plant cells to selectively digest the AP IM but not the hybrid AP–vacuole fusion membrane. Therefore, the glycosylated proteins obtained from endosomes and lysosomes or vacuoles onto the hybrid membrane may confer this protection from hydrolysis.

(8) How does the hybrid AP–lysosome/vacuole fusion membrane reconstruct or regenerate after fusion? We concluded that the vacuole required to remove its membrane from the AP–vacuole fusion hybrid structure with surface area was at least twice as that of the vacuole surface area (Figure 5). The spatiotemporal changes in AP–lysosome/vacuole fusion are currently unclear. Therefore, spatiotemporal reconstruction or regeneration after fusion, such as which part of the membrane would be used for reformation and what format would be reformed, remains a mystery. Nevertheless, in mammalian cells, lysosomes are reformed from ALs by the autolysosome reformation (ALR) process through the reactivation of mammalian target of rapamycin (mTOR) when autophagy is terminated [46], and AP components are recycled by the ACR complex [47,48]. Although the study on ACR evaluated STX17 and ATG9A, it did not clarify whether other AP protein components and membrane lipids are recycled [47,48]. The transient process by which the AP–lysosome hybrid membrane releases AP components through ACR and regenerates ALs remains unclear. Furthermore, the literature does not clarify whether lysosomes generated from ALs will be used directly or fused with old lysosomes to obtain their functions.

In contrast to the multiple small lysosomes in mammalian cells, yeast and plant cells only contain one to a few large vacuoles. Thus, the reformation of the hybrid AP–vacuole membrane in these cells is quite different from that of the ALR in mammalian cells. No previous reports have described the presence of a hybrid AP–vacuole reformation process generating new vacuoles in yeast and plant cells. However, as we mentioned above, the total surface area of filled ABs in a vacuole is 2–4-fold the surface area of that vacuole when the vacuole volume is filled to about 50% by ABs. Thus, the fused OMs of the APs are not continuously used to expand the vacuole surface area. They must be partially and dynamically removed by protrusion into the cytosol and/or invagination into the vacuolar lumen to bud off for further degradation or recycling. Vacuolar invagination has been observed and studied in detail during autophagy induction, depending on starvation and the autophagy pathway through microautophagy. Furthermore, the smooth areas on the vacuolar membranes were used as precursors of tubular invaginations, and a higher density of transmembrane particles existed in the more basal areas of the tube and were absent at the tips [50], which may have been associated with vesicle budding at the tips. We also observed vacuolar invaginations by time-lapse microscopy. In addition, we observed vesicle-like structures budding off and fusing into the vacuolar membrane, and fusion and fission among vacuoles during autophagy induction (unpublished data). However, we do not have many details regarding these processes, their implications, or their quantitative contributions to changes in vacuole size and volume. The spatiotemporal regulation of lysosome/vacuole regeneration has not yet been fully identified. What are the exact signals that trigger lysosome/vacuole regeneration? Is the site to generate new lysosomes or remove vacuolar components selective? What is the signal other than nutritional depletion that terminates lysosome/vacuole regeneration? These questions need to be answered more precisely in the future. Vacuole regeneration in plant cells during autophagy has not been reported in the literature. The similarities and differences in lysosome/vacuole regeneration between mammalian and yeast and plant cells require further investigation.

**Figure 6:**
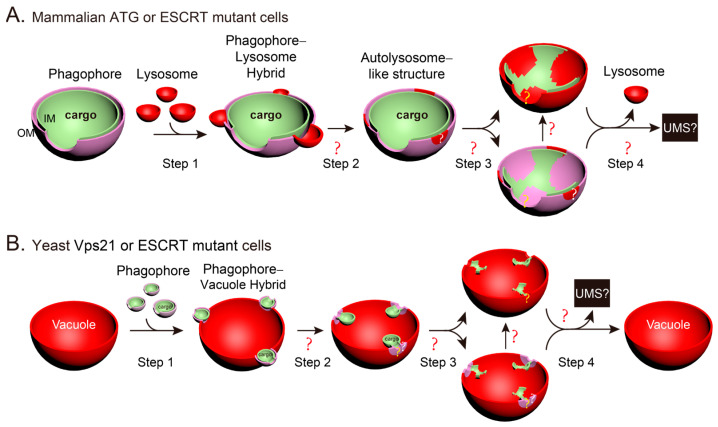
Three-dimensional (3D) illustrations showing the spatiotemporal changes in membranes during and after phagophore–lysosome/vacuole fusion. The drawings are based on previous studies showing that phagophores can fuse with lysosomes in mammalian cells [43,44] and with vacuoles in yeast cells [41], and the summarized 2D models were presented in a previous study [70]. This figure is similar to Figure 4, except that it shows phagophores with open holes instead of closed APs. (**A**) In mammalian ATG or endosomal sorting complex required for transport (ESCRT) mutant cells, multiple lysosomes fuse with a phagophore to degrade the phagophore and its cargoes. (**B**) In yeast Vps21 or ESCRT mutant cells, multiple phagophores fuse with a vacuole to degrade the phagophores and their cargoes. The unclear or uncertain processes are indicated with “?” marks.

(9) How are the fusion and regeneration of the phagophore–lysosome/vacuole realized? The AP–lysosome/vacuole fusion should be the most prominent format during autophagy. Some researchers thought that phagophores (unclosed APs) would not fuse with lysosomes/vacuoles because they assumed the cytosolic contents inside phagophore IMs would be released into the cytosol through the phagophore hole after phagophore–lysosome/vacuole fusion. This assumption came from a simple 2D understanding of a complex 3D biological process because these researchers believed that the phagophore outer and inner membrane would finally have the same curvature after phagophore–lysosome/vacuole fusion. However, the fusion of phagophores with lysosomes/vacuoles has been reported in both mammalian and yeast cells [41,43,44]. Mammalian phagophores fuse with lysosomes at a slower rate than AP–lysosome fusion, and phagophore IMs mixed with lysosomal proteins become resistant to degradation by lysosomal enzymes [43,44]. Therefore, the impossibility of phagophore–lysosome/vacuole fusion assumed in a simple 2D understanding is not valid [70]. To better understand phagophore–lysosome/vacuole fusion, we described the most probable situations in 3D (Figure 6). Lysosomes should be able to fuse with both APs and phagophores unless there are strict signals to block the fusion between phagophores and lysosomes but allow the fusion between APs and lysosomes. STX17 was found to be recruited to more negatively charged mature APs by electrostatic interactions [57,58] to trigger fusion in mammalian cells [31]. However, the phagophores in mammalian mutant cells can also acquire STX17 and fuse with lysosomes [43,44]. Therefore, evidence for such a signal or situation is currently not available (Figure 6A). We agree that some questions regarding lysosome–phagophore fusion remain unanswered. In addition to the common unanswered questions regarding lysosome–AP fusion, specific questions related to phagophores during lysosome–phagophore fusion, such as the spatiotemporal changes in phagophore holes during and after fusion, also remain unanswered. For phagophore–lysosome fusion in mammalian cells, the relevant questions include whether the phagophore hole is maintained when the phagophore IM with its cytosolic contents are digested by lysosomal hydrolases and whether the mammalian phagophore hole on the phagophore OM would finally close, and if yes, when and how? For phagophore–vacuole fusion in yeast, the most unclear issue is how the phagophore IM is separated from the hybrid phagophore–vacuole fusion membrane to release unclosed ABs into the vacuolar lumen [41], whether the phagophore OM hole on the phagophore–vacuole hybrid membrane closes, and how the hole on the phagophore IM is maintained after fusion (Figure 6B). To date, there is no information on vacuole regeneration from hybrid phagophore–vacuole fusion membranes. Therefore, it is unknown how the additional membranes are removed from the hybrid membrane.

## 7. Possible Resolutions for the Unknown Aspects During and After AP–Lysosome/Vacuole Fusions

On the basis of their identities, unclosed and closed APs or APs and ALs are distinguished using different techniques such as biochemical assays or fluorescence observations [44,52,53,71,72,73]. The differences between the outer and inner membranes of APs and the fusion process of the AP OM with lysosome/vacuole membranes are difficult to observe using fluorescence microscopy. However, the lysosome contents existing only in the space between the outer and inner membranes but not in the AP lumen immediately after fusion were deduced with time-lapse fluorescence microscopy as a ring-shaped lysosomal marker that first appeared at the AP membranes and then diffused into the AP lumen. The ring-shaped lysosomal signals last longer in mutant cells that generate phagophores [43,44]. Techniques to clearly distinguish and track changes in the outer and inner membranes of APs during fusion and regeneration in real-time are lacking.

TEM or immuno-EM partially provided information on the outer and inner membranes of APs based on their morphology and specific markers. LAMP1 signals were detected on the OM, but not the IM, in wild-type cells, and in both the OM and IM of the phagophores in CHMP2A-depleted cells [44]. Cryo-FIB and cryo-ET have greatly improved the identification of membrane features. However, different membrane features during AP–lysosome/vacuole fusion and regeneration cannot be completely resolved by these methods because of the limited number of membrane feature markers; the static, not dynamic, nature of the observations; and the limited availability of gold-labeling markers for immuno-EM. In the future, identification of AP membrane features at different stages, especially for the outer and inner membranes of APs, should be prioritized. Sequential TEM in combination with immuno-EM using more protein labeling markers in the same sample may provide more information. Furthermore, AP–lysosome/vacuole fusion and regeneration occur in a spatiotemporal pattern, and 3D dynamic observations and reconstruction are required to better understand these processes. The final solution relies on the emergence of better real-time observation techniques in 3D with high resolution.

## 8. Concluding Remarks

The current understanding of membrane features during the AP–lysosome/vacuole fusion and regeneration processes is scarce. The complete AP–lysosome/vacuole fusion process has not been sequentially characterized in live cells because of the lack of distinguishable markers for the outer and inner membranes of APs and the limited resolution of fluorescence microscopy. ACR and ALR have been detected in mammalian cells. However, our understanding of the spatiotemporal regulation of these two processes remains limited, and the mechanism by which phagophores fuse with lysosomes/vacuoles remains unclear. Complete characterization of the AP–lysosome/vacuole fusion and regeneration steps in both mammalian and yeast/plant cells remains a big challenge (Figure 4), and is heavily reliant on the availability of new and better techniques.

## Data Availability

Not applicable.
